# Targeted Next Generation Sequencing Revealed Novel Variants in the *PKD1* and *PKD2* Genes of Iranian Patients with Autosomal Dominant Polycystic Kidney Disease

**DOI:** 10.34172/aim.2022.95

**Published:** 2022-09-01

**Authors:** Maryam Hosseinpour, Fariba Ardalani, Marzieh Mohseni, Maryam Beheshtian, Sanaz Arzhangi, Shahrzad Ossareh, Hossein Najmabadi, Ali Nobakht, Kimia Kahrizi, Behrooz Broumand

**Affiliations:** ^1^Genetics Research Center, University of Social Welfare and Rehabilitation Sciences, Tehran, Iran; ^2^Division of Nephrology, Department of Medicine, Hasheminejad Kidney Center, Iran University of Medical Sciences, Tehran, Iran; ^3^Department of Nephrology, School of Medicine, Shahid Beheshti University of Medical Sciences, Tehran, Iran; ^4^Pars Advanced and Minimally Invasive Medical Manners Research Center, Pars Hospital, Tehran, Iran

**Keywords:** Autosomal dominant, Iranian families, Next generation sequencing, PKD1, PKD2, Polycystic kidney disease, Variants

## Abstract

**Background:**

Autosomal dominant polycystic kidney disease (ADPKD), one of the common inherited disorders in humans, is characterized by the development and enlargement of renal cysts, often leading to end-stage renal disease (ESRD). In this study, Iranian ADPKD families were subjected to high-throughput DNA sequencing to find potential causative variants facilitating the way toward risk assessment and targeted therapy.

**Methods:**

Our protocol was based on the targeted next generation sequencing (NGS) panel previously developed in our center comprising 12 genes involved in PKD. This panel has been applied to investigate the genetic causes of 32 patients with a clinical suspicion of ADPKD.

**Results:**

We identified a total of 31 variants for 32 individuals, two of which were each detected in two individuals. Twenty-seven out of 31 detected variants were interpreted as pathogenic/likely pathogenic and the remaining 4 of uncertain significance with a molecular diagnostic success rate of 87.5%. Among these variants, 25 *PKD1/2* pathogenic/likely pathogenic variants were detected in 32 index patients (78.1%), and variants of uncertain significance in four individuals (12.5% in *PKD1/2*). The majority of variants was identified in *PKD1* (74.2%). Autosomal recessive PKD was identified in one patient, indicating the similarities between recessive and dominant PKD. In concordance with earlier studies, this biallelic *PKD1* variant, p.Arg3277Cys, leads to rapidly progressive and severe disease with very early-onset ADPKD.

**Conclusion:**

Our findings suggest that targeted gene panel sequencing is expected to be the method of choice to improve diagnostic and prognostic accuracy in PKD patients with heterogeneity in genetic background.

## Introduction

 Autosomal dominant polycystic kidney disease (ADPKD) is the most frequent monogenic kidney disorder with an estimated frequency varying from 1/400 to 1/1000 in the general population.^1^ Multiple renal cysts, as the major presentation, invoke enlarged and irregular-shaped kidneys. ADPKD primarily associates with the *PKD1* gene [MIM#601313, chromosome 16p13.3] and/or the *PKD2* gene [MIM#173910, chromosome 4q21] mutations,^2,3^ ascribing 85% and 15% of ADPKD patients, respectively.^4^ Stronger association with a severe clinical presentation and poorer prognosis have been reported for mutations in *PKD1* than those in the *PKD2* gene, suggestive of the critical role of genes in outcome prediction of patients with ADPKD.^4^

 Due to the allelic heterogeneity, the large size, and complex genomic structure of *PKD1* and *PKD2*, andlack of hotspot site for mutations in these two genes, the genetic analysis of ADPKD is challenging.^5^ *PKD1* is a complex and large gene that spans 46 exons encoding polycystin-1 with 4303 amino acids. *PKD2* contains 15 exons and encodes polycystin-2 consisting of 968 amino acids. Six homologous genes (pseudogenes) on chromosome 16 show 97.7% identity with exons 1 to 33 of *PKD1* gene in sequence, carrying larger deletions in comparison to *PKD1*.^6,7^ In patients with mutations in *PKD2*, a milder clinical course compared to *PKD1 *patients, fewer renal cysts and milder hypertension (HTN) lead to delayed progression to end-stage kidney failure.^8^ A high level of allelic heterogeneity has been observed among ADPKD patients, with over 2300 and 270 germline variants reported to date in *PKD1* and *PKD2*, respectively in the Autosomal Dominant Polycystic Kidney Mutation Database (PKDB; http://pkdb.mayo.edu/), excluding our most recent data. This database has also recorded 9 somatic mutations of the *PKD1* and 27 somatic mutations of the *PKD2* gene.

 In case of negative family history in patients with atypical clinical or imaging features, the genetic diagnosis of ADPKD is beneficial to identify a familial donor for renal transplantation, guiding patient management and treatments, and improving the precision of genetic counseling and anticipating disease prognosis based on the PROPKD score.^9-11^

 In this study, we aimed to use targeted next generation sequencing (NGS) to study the mutational spectra of the *PKD1* and *PKD2* genes in 32 Iranian patients with ADPKD. In addition, we reviewed and compared the mutational spectrum of the *PKD1* and *PKD2* genes, in both the Middle East and Iranian populations.

## Materials and Methods

###  Family Ascertainment and Clinical Diagnosis

 In this study, 32 unrelated ADPKD probands (16 males and 16 females) presenting classical dominant inheritance pattern were recruited from nephrology clinics in Tehran, Iran. Pei’s ultrasound diagnostic criteria^12^ were applied to diagnose ADPKD in the selected probands. In patients older than 40 years, we ruled out ADPKD if their ultrasound showed absence of kidney cysts. Our investigated probands had one affected parent with ADPKD. All subjects either had at least one family member with a more serious disease presentation (end‐stage renal disease; ESRD) before the age of 60 years or they had already manifested ESRD. The study was approved by the Institutional Ethics Board at the University of Social Welfare and Rehabilitation Sciences (USWR), Tehran, Iran. Prior to recruiting to the study, informed consent was taken from patients. Subsequently, we extracted DNA from peripheral blood sample of the probands using the standard salting out protocol.^13^ The quality of DNA samples was analyzed by nanodrop (Thermo 2000).

###  Targeted Next Generation Sequencing Data Analysis 

 All coding sequences and intron–exon boundaries of genes of interest were captured using solution-based biotinylated oligonucleotide custom-designed probes. The hybridized DNAs with biotinylated probes were bound to streptavidin-coated magnetic beads and then in a magnetic field, they were isolated from the rest of the genomic DNA. For each sample, the captured regions were bar-coded using short oligonucleotides, and were pooled and subjected to paired-end sequencing for 150 cycles (yielding 150 bp reads) on Illumina NextSeq.

 We tested 63 samples and the mean depth of coverage was 395x (range 199x – 799x). Table S1 lists the genes included in our panel (see Supplementary file 1).

###  Bioinformatics Analysis

 Using Burrows-Wheeler Aligner, we aligned generated sequence reads for each sample to the reference human genome. Identification of genetic variations comprising SNP or insertion-deletion (indel) was done by application of the HaplotypeCaller module of the GATK package. We used regions with at least 20-fold depth of coverage for calling variants. A nucleotide that differed from the reference sequence in at least 25% of the reads aligned to a given position was considered as a variant.

 Common variants were filtered against Genome Aggregation Database (http://gnomad.broadinstitute.org) and Iranome database (http://iranome.com). We validated all potentially causal variants identified in targeted-NGS by Sanger sequencing using the ABI PRISM Big Dye Terminator Cycle Sequencing Kit and ABI PRISM 3130 Genetic Analyzer (Applied Biosystems, Foster City, CA, USA). Additionally, we sequenced siblings and parents, when available, to confirm co-segregation of the variant within the family. The significance of missense variants was assessed using the ADPKD Mutation Database (https://pkdb.mayo.edu/), Varsome (https://varsome.com/), SIFT (https://sift.bii.a-star.edu.sg/), PolyPhen2 (http://genetics.bwh.harvard.edu/pph2/) and Mutation Taster (http://www.mutationtaster.org/), as previously described.^14-16^ Variants were scored using the American College of Medical Genetics (ACMG) variant interpretation guideline.^17^ The variants’ predicted effects on splicing were assessed using:

 BDGP (http://www.fruitfly.org/seq_tools/splice.html),^18^

 Spliceport (http://spliceport.cs.umd.edu/),^19^

 GeneSplicer (http://www.cbcb.umd.edu/software/GeneSplicer/gene_spl.shtml).^20^

## Results

 In the present study, we performed genetic diagnostics for 32 unrelated ADPKD families consisting of 16 males and 16 females mainly from Persian and Azeri ethnical groups (Table 1). The clinical characterization of the suspected ADPKD patients is summarized in Table 1. A positive family history was present in 90.6% (29/32) of patients. Mean age was 51 years (range: 27 to 84 years).

**Table 1:** Clinical Characterization of Suspected ADPKD Patients.

**Patient Code**	**Sex**	**Ethnicity**	**Age (year)**	**Age at onset (year)**	**No. of Affected Individuals in Family**	**Renal Phenotype**	**History Urolithiasis**	**History HTN**	**Liver Cyst**	**Vascular abnormalities**	**CKD**	**ESRD<60 year**	**Renal transplantation (age/year)**
56120	M	Persian	66	33	24	Enlarged kidneys, polycystic kidney, left kidney transplantation	N	Y	N	N	Y	Y	Y
56160	M	Persian	46	35	14	Polycystic kidney	N	Y	N	N	Y	N	N
56180	M	Persian	55	30	10	Polycystic kidney	N	Y	N	N	Y	Y	Y
56200	F	Lur	43	30	3	Parenchymal cyst, up to 47	N	Y	N	Y	N	N	N
56220	M	Azeri	38	35	1	Enlarged kidneys, polycystic cortical, cyst with calcification, up to 135*15 mm(R)141*12 mm(L)	Y	Y	N	N	N	N	N
56460	F	Persian	40	19	5	Polycystic kidney	N	Y	Y	N	N	N	N
56480	M	Kurd	84	65	0	Enlarged kidneys, parenchymal cyst	N	N	N	N	N	N	N
56500	F	Azeri	62	34	9	Polycystic kidney	N	Y	N	Y	Y	Y	Y (25 year)
56580	M	Persian	51	25	9	Polycystic kidney	Y	Y	Y	N	Y	Y	N
56600	F	Persian	66	24	5	Polycystic cortical and medullary, up to 16*15 mm(R)10*9 mm(L)	N	Y	Y	N	N	N	N
56640	M	Persian	63	20	7	Polycystic kidney	N	Y	N	Y	Y	Y	Y (20 year)
56680	F	Persian	55	26	7	Enlarged kidneys, Polycystic up to 90 mm	Y	Y	N	N	Y	Y	Y (32 year)
57020	F	Azeri	43	26	2	Enlarged kidneys, polycystic up to 35, Distort both renal architecture	Y	Y	N	N	Y	Y	Y (20 year)
57060	M	Persian	33	16	2	Left kidney enlarged, both kidney multiple cyst	Y	Y	N	N	N	N	N
57080	M	Azeri	36	6	1	Polycystic up to 21	Y	Y	Y	Y	Y	N	N
57140	M	Lur	57	30	3	Polycystic cortical, up to 14 mm(R)*18 mm(L)	N	N	Y	N	Y	N	N
57180	M	Persian	27	25	11	Small cyst 28*27 mm(R) 21*12 mm(L)	N	N	N	Y	N	N	N
57220	F	Azeri	58	35	1	Polycystic cortical, up to 59*33 mm(R)70*45 mm(L)	Y	Y	N	N	N	N	N
57280	M	Persian	59	28	2	Polycystic, up to 83*56 mm*56 mm(R)*44 mm(L)	Y	Y	N	Y	Y	N	N
57340	F	Persian	64	40	1	Enlarged kidneys, parenchymal cyst up to 46*46 mm a kidney transplant	Y	Y	Y	N	Y	Y	Y (50 year)
57360	F	Persian	48	25	6	Corticomedullary cyst, up to 61 mm (R)59 mm (L)	N	Y	N	Y	N	N	N
57380	F	Azeri	56	35	11	Polycystic, up to 29*22 mm(R)49*44 mm(L)	N	Y	N	N	N	N	N
57400	F	Persian	35	35	5	Enlarged kidneys 160 mm (R)160 mm (L), polycystic up to 40mm(R) 60 mm(L)	Y	N	N	N	N	N	N
57460	M	Persian	69	24	5	Polycystic kidneys	Y	Y	Y	Y	Y	Y	Y
57500	F	Azeri	63	32	6	Enlarged kidneys, polycystic, up to 20 mm	Y	Y	Y	N	N	N	N
57540	F	Gilaki	43	26	2	Enlarged kidneys 180 mm, polycystic	Y	Y	Y	N	N	N	N
57580	M	Gilaki	41	38	0	Polycystic kidney	Y	Y	Y	Y	Y	N	N
57620	F	Persian	61	50	3	Enlarged kidneys, polycystic, Distort both renal architecture	N	Y	N	N	Y	Y	Y (36 year)
57640	M	Lur	60	22	7	Polycystic kidney	Y	N	Y	N	N	N	N
57860	F	Persian	57	46	0	Enlarged kidneys 129*54 mm(R)132*57 mm(L), polycystic cortical, up to 32*28 mm(R)24*22 mm(L)	Y	Y	N	N	N	N	N
57900	M	Persian	37	35	3	Small parenchymal Cyst contain milk calcium foci	Y	N	N	Y	N	N	N
58040	F	Azeri	41	28	8	Enlarged kidneys 136 mm(R)135 mm (L), Polycystic cortical, up to 47*35 mm(R)54*40 mm(L)	Y	Y	Y	Y	N	N	N
58040	F	Azeri	41	28	8	Enlarged kidneys 136 mm(R)135 mm (L), Polycystic cortical, up to 47*35 mm(R)54*40 mm(L)	Y	Y	Y	Y	N	N	N

M, Male; F, Female; Y, Yes; N, No; CKD, Chronic kidney disease; ESRD, end-stage renal disease

 We identified a total of 31 variants for 32 individuals, two of which were each detected in two individuals. According to the ACMG guideline, for 27 individuals, we detected pathogenic/likely pathogenic variants and the remaining 4 of uncertain clinical signiﬁcance with a diagnostic rate 87.5%. A total of 14 novel variants were detected in 14 individuals.

 We detected pathogenic and likely pathogenic variants in 27 out of 32 patients carrying a total of 31 different mutations, 23 in *PKD1*, 6 in *PKD2*, and 2 in *PKHD1* (Supplementary file 2, Table S2). Among all the detected variants, there was a total of 14 (45.2%; 14/31) novel gene variants, not previously linked to PKD in the literature (Table S2). All detected variants were confirmed by the Sanger sequencing method.

 Thirty-one different mutations were observed in the 32 affected individuals, of which 12 were missense mutations (38.7%), 10 frameshift mutations (32.2%), 6 stop gain mutations (19.3%), and 3 splice-site mutations (9.7%) (Table S2). The variations were distributed throughout the entire gene, more predominantly at the 5’ end of the *PKD1* gene compared to the 3’ end of this gene. A missense and a splice site variant in *PKHD1* also were identified in one of our patients (Table S2). Based on the guideline from the ACMG, the variants were classified as follow: a total of 17 variations (9 frameshift, 5 stop gain, and 3 splice site) as pathogenic, 10 variants (8 missense, 1 stop gain, and one frameshift) as likely pathogenic, and the remaining 4 as of uncertain significance. Mutations mostly occurred in exon 15 (n = 5), and 5, 29, and 34 (each 2 variants) of the *PKD1* gene, and exon 3 of the *PKD2* gene (n = 2) (Table S2). All variants detected in our investigated patients are presented in Table S2.

 The *in silico* pathogenicity predictions for each variant using, SIFT, PolyPhen, and Mutation Taster software are shown in Table S2. According to ACMG, the variants for 4 patients were of uncertain significance including c.692T > C, c.5528G > T, and c.9698A > T in *PKD1* gene, and c.2186T > A in *PKD2*. In “Mutation Taster”, all these 4 variants were disease causing.

## Discussion

 In the clinical management of ADPKD, genetic diagnosis testing is not pervasive. The segmentally duplicated region in the *PKD1* gene has been considered as a major obstacle to genetic analysis in ADPKD.^21^ Other complicating factors include extreme genetic and allelic heterogeneity, difficult clinical interpretation due to non-truncating alleles and the possibility of hypomorphic low-penetrance variations.^21^ On the other hand, genetic testing is increasingly applied as an indispensable component for diagnosis, prognosis and choosing treatment options.^21^ Many different methods have been applied, such as conventional sequencing, and whole exome sequencing, all of which have their limitation due to the complexity of these genes. Here, we applied the targeted-NGS method in our investigated ADPKD patients.

 Mutations of the *PKD1* and *PKD2* genes affect about 85% and 15% of ADPKD cases, respectively.^22^ In concordance with other studies and the PKDB database, these rates were 74.2% (23 out of 31 detected variants) in *PKD1 *and 19.3% (6 out of 31 detected variants) in *PKD2 *in our study. Using the NGS technique, Ranjzad et al reported a mutation rate of 88.9% and 11.1% in *PKD1* and *PKD2*, respectively, in 18 Iranian ADPKD patients.^23^ Application of a custom designed Nimblegen chip, capturing the *PKD1* and *PKD2* genes, followed by NGS, all of mutations found in the study by Bitarafan and Gharshasbi were located in the *PKD1* gene, with a lack of mutation in *PKD2*.^24^ Hoefele et al in their routine molecular testing of 93 ADPKD patients using long-range PCR and direct sequencing, reported 86.7% *PKD1 *and 13.3% *PKD2 *mutations from Germany.^25^ In France, Audrézet et alinvestigated 700 unrelated patients with ADPKD using direct sequencing, followed by quantitative fluorescent multiplex polymerase chain reaction or array-comparative genomic hybridization and reported mutation rates of 83.8% and 16.2% in *PKD1* and *PKD2*, respectively.^26^ Investigation of 62 unrelated Han Chinese families by targeted NGS revealed mutation rates of 84.2% and 15.8%.^27^ In South Korea, among 20 unrelated ADPKD patients using LR-PCR and direct sequencing, the mutation rates were 83.3% and 16.7% in *PKD1* and *PKD2*, respectively.^28^

 In terms of the effect of the type of mutations on protein production, 18 out of 31 identified variants cause ADPKD through loss of polycystin-1 and polycystin-2 proteins. The majority of changes in our patients were truncating (because of nonsense changes, frame-shift deletion and/or insertion, or splice defects), which is in concordance with previous studies.^21^ No large gene deletions or repetitions were detected in our patients.

 Overall, 14 novel variants were identified and 17 variants were previously reported as disease causing (Table S2), suggestive of the high level of allelic heterogeneity in these genes. Eight novel pathogenic and likely pathogenic variants were detected in the *PKD1* gene. A heterozygous likely pathogenic missense variant c.692T > C (p.Leu231Pro) was identified in exon 5 of *PKD1*, in a 40-year-old female who presented with polycystic kidney, arterial HTN, and liver cyst (Table 1 and Table S2). This variant in the *PKD1* gene changes a conserved Leucine amino acid into a Proline at the position 231, which are different in size, charge, and hydrophobicity-value. The variation is located within a WSC domain, a putative carbohydrate binding domain, which can potentially disturb this domain and abolish its function.^29^ In another female with urolithiasis, HTN, chronic kidney disease (CKD), ESRD before age 60, and renal transplantation at 54 years of age, we identified a novel heterozygous 1-bp duplication (c.1938dupG) in exon 10 of the *PKD1* gene, resulting in frameshift and premature termination of p.(Cys647ValfsTer67), which was predicted to be pathogenic by three predicting tools (Table 1 & Table S2). Another novel variant (c.2534T > A, p.Leu845X) was detected in *PKD1*, predicted to be pathogenic by three predicting tools (Table 1 and Table S2).

 NGS testing was informative and provided supporting evidence in favor of ADPKD diagnosis in the proband (57580). This variant is a Thymine to Adenine substitution at position 2534 in exon 11 of *PKD1*, causing a stop gain at codon 845 (Table 1 and Table S2).

 In addition, we identified three novel variants in exon 15 of *PKD1* including c.6759delC (p.Glu2254SerfsTer60), c.4625T > G (p.Val1542Gly), and c.5528G > T (p.Gly1843Val) (Table 1 and Table S2).

 The first one (c.6759delC), which is predicted to be pathogenic by ACMG criteria and disease-causing by Mutation Taster, is a heterozygous mutation causing a frameshift at amino acid 60, leading to a stop codon at codon 2314. This variant was detected in an individual with urolithiasis, HTN, liver cyst, CKD, and ESRD before 60 years of age (Table 1 and Table S2).

 The second variant (c.4625T > G) was a likely pathogenic missense variant detected in a 66-year-old female presenting with liver cyst and HTN. This variant was suggested to be damaging and probably damaging by PolyPhen and SIFT, respectively. This variant, which has been located within a PKD domain 10, led to the change of a Valine amino acid into a Glycine. It has been predicted that any change in this position can disturb this domain and abolish its function due to the flexibility of Glycine, which might disturb the required rigidity of the protein at this position.^29^

 The third variant, c.5528G > T (p.Gly1843Val), in exon 15 *PKD1*, was a heterozygous uncertain significance missense variant, identified in a 33-year-old male, with urolithiasis, kidney enlargement, multiple cysts in both kidneys and HTN phenotype. The mutant residue is more hydrophobic and bigger than the wild-type one. It has been suggested that flexibility of glycine at this position might be necessary for the protein’s function.^29^

 A novel pathogenic variant c.8005delG was also identified in *PKD1* in a 41-years old individual with urolithiasis, HTN, and liver cyst phenotype. This variant is a single nucleotide deletion (delG) at position 8005 in exon 21 of *PKD1*, causing a frameshift at amino acid 2669, which leads to a stop codon at 16 residues later. This mutation has not been reported in 1000 Genome, gnomAD, and ESP6500 databases, and was predicted to be pathogenic by ACMG classification (Table 1 and Table S2).

 In exon 30 of *PKD1*, we found another novel likely pathogenic heterozygous missense variant, c.10032C > G (p.Phe3344Leu) in an individual with HTN, CKD, ESRD before 60 years of age, and renal transplantation at age 36. PolyPhen predicted this variant as probably damaging. It has not been reported in the 1000 Genome, gnomAD and ESP6500 databases (Table 1 and Table S2). Compared to the conserved wild-type, the mutant residue is smaller with different charge and hydrophobicity-value which might affect contacts with the lipid-membrane.^29^

 We identified another novel variant in exon 31 of *PKD1* (c.10159delC, p.His3387ThrfsTer10), which was a single nucleotide deletion (delC) at position 10159, causing a frameshift at codon 3387. This pathogenic variant was detected in a 64-year-old symptomatic female with a possible diagnosis of ADPKD. This female suffered from enlarged kidneys, parenchymal cyst, urolithiasis, HTN, liver cyst, CKD, ESRD before age 60, and renal transplantation at age 54 years. This mutation was predicted to be disease-causing by Mutation Taster (Table 1 and Table S2).

 Finally, we detected the novel heterozygous pathogenic variant c.12624_12625dupTG (p.Glu4209ValfsTer150) in exon 46 of *PKD1* in an individual suspected of ADPKD. This variant is two single nucleotide duplications (dupTG) at position 12624_12625 of *PKD1*, causing a frameshift at codon 4209. The predicting tools provided strong evidence of pathogenicity of this frameshift variant. In addition, this mutation has not been described in the 1000 Genome, gnomAD, and ESP6500 databases. This variant was found in a 59-years-old male who had urolithiasis, HTN, vascular abnormalities, and CKD (Table 1 and Table S2).

 Furthermore, a novel likely pathogenic variant c.249_250dupCC (p.Arg84ProfsTer34), an uncertain significance variant c.2186T > A (p. Leu729Gln), and a pathogenic variant c.2522+1G > A were detected in exon 1, exon 11, and intron 13 of the *PKD2* gene, respectively. The c.249_250dupCC variant is a 2-bp duplication cytosine at positions 249_250 exon 1 of the *PKD2* gene, resulting in frameshift and premature termination of p.Arg84ProfsTer34, and it was predicted to be disease-causing by Mutation Taster. The other *PKD2* missense variant (c.2186T > A, p.Leu729Gln), was detected in an 84-year-old male who presented with enlarged kidneys, and parenchymal cyst (Table 1 and Table S2). Compared to the wild-type, the mutant residue, which is located at the very conserved α-helix region, is bigger, while the wild-type residue is more hydrophobic than the mutant. It has been suggested that any change at this region might affect the secondary structure.^29^ Finally, the last *PKD2* c.2522+1G > A splice donor variant was identified in intron 13 of *PKD2*, in a 43-year-old female who presented with parenchymal cyst, and HTN. The predicting tools provided strong evidence of pathogenicity of this splice site variant (Table 1 and Table S2).

 One of our patients was *PKD1*/*2*-negative, found to have an alternative genetic diagnosis as autosomal recessive PKD. In this patient, we identified biallelic mutations in *PKHD1* including the missense variant c.4870C > T (p.Arg1624Trp) in exon 32 and the splice acceptor variant c.10157-1G > A in intron 61 (Table 1 and Table S2).

 Overall, the most commonly detected pathogenic alleles were c.9829C > T (p.Arg3277Cys), and c.10423C > T (p.Gln3475X) in *PKD1*, which were each seen in two patients (Table S2).

 Biallelic *PKD1 *or *PKD2* pathogenic variants have been reported in ADPKD patients with very early-onset of symptoms.^30^ We have found biallelic *PKD1* reported variant p.Arg3277Cys in a 43-year-old Azeri female whose age of onset was 26 (Table 1 and Table S2). Furthermore, in concordance with the study by Hopp et al,^31^ we found that the p.Arg3277Cys variant causes a rapidly progressive disease in this individual. She was homozygote for the p.Arg3277Cys variant, showing the most severe features among our patients, with enlarged kidneys, up to 35 cysts, distorting both renal architecture, urolithiasis, HTN, CKD, ESRD before age 60, and requiring renal transplantation at 20 years of age. The heterozygous p.Arg3277Cys variant in the *PKD1* gene leads to just a few cysts or no evidence of disease,^32^ which is compatible with our finding in a heterozygote carrier 27-year-old male with small cysts and vascular abnormalities without any other features (Table 1 and Table S2). Compared to the patients with *PKD2*-related ADPKD, *PKD1*-related ADPKD cases often present with remarkably larger kidneys with more cysts^32,33^ as Table 1 shows, this was the case in our patients, with larger kidneys seen in *PKD1*-related ADPKD patients.

 Two major issues (the 20-year earlier occurrence of ESRD in patients with *PKD1* than those with *PKD2 and the association between *the position of the *PKD1* mutation and the onset age of ESRD) have been underscored in genotype-phenotype correlation studies.^33^ The mean age of ERSD onset due to *PKD1* variations is about 20 years earlier than that of *PKD2* mutations.^33^ None of our patients with *PKD2* variants showed ESRD before 60 years of age; however, 40% of our patients carrying *PKD1* variants presented ESRD before 60 years of age. All of our *PKD2*-related ADPKD patients also had a milder phenotype and had a maximum of two additional clinical problems reported in Table 1, other than kidney cysts.

 In our study, pathogenic truncating variants in *PKD2* were reported to be linked to more severe disease than non-truncating ones, which is compatible with other studies.^34^

 Among 115 distinct variants of *PKD1* found in the Middle East region in ADPKD patients, 45 (39%) were previously unreported, conﬁrming broad allelic heterogeneity (Supplementary file 3, Table S3). The most prevalent mutations reported from this region include p.Arg1672fs97X (c.5014_5015delAG) in exon 15 (n = 3) from Iran, Kuwait, and Oman, and p.Gln3475Ter (c.10423C > T) in exon 34 from Iran (n = 3). The p. Gln2243Ter (c.6727C > T) variant in exon 15, p.Glu2810Ter (c.8428G > T) in exon 23, p.Leu4137Pro (c.12410T > C) and p.Val4146Ile (c.12436G > A) in exon 45, were identified twice in Iranian, Saudi Arabians and Kuwaitis. Among *PKD1* mutations reported from Iran, most occurred in exons 15, and 5, 34, 44, and 45, which is compatible with Kuwaitis’ *PKD1* mutations. The *PKD2* mutations most presented in exons 1, 3, and 4 in Iranian patients with ADPKD.

 One of our patients was heterozygote for two mutations in *PKHD1* including the missense variant p.(Arg1624Trp); c.4870C > T in exon 32 and the splice acceptor variant c.10157-1G > A in intron 61, suggesting the similarities between recessive and dominant PKD. Edrees et al also found this mutation in the *PKHD1* gene among 8 out of 18 PKD Saudi patients, suggesting that mutations in this gene may be more prevalent than that of the *PKD1* and *PKD2* genes among Saudi patients.^10^ Consideration of ARPKD as a differential diagnosis in adult PKD with a negative family history has been emphasized by the finding of biallelic *PKHD1* missense variants.

 Information from family history with ultrasound imaging results are used to diagnose ADPKD nowadays.^22^ Manifestation of three or more renal cysts (unilateral or bilateral) in individuals aged 15–39 years is sufficient to establish the diagnosis. However, for individuals aged 40–59 years and individuals over 60 years, two or more cysts and four or more cysts, respectively, in each kidney are required for diagnosis.^22^ Genetic testing can provide a definitive diagnosis before the age of onset, or in cases with no kidney cysts and negative family history, in addition to disease progression and prognosis.^22,35^ All Our patients except two had a family history of the disease, ranging from 1 to 24 patients in the family, including both unilateral and bilateral cysts.

 In summary, the NGS advancements not only allow the analysis of several genes in a single set with relatively low costs, but also provide a powerful method to diagnose genetically heterogeneous diseases such as PKD with a broad phenotypic spectrum.

 In conclusion, genetic diagnosis enables us to detect ADPKD phenocopies and avoid potentially harmful treatment in* PKD1*/*2*-negative cases. Our study emphasizes the role of genetic heterogeneity in PKD, leading us to infer that personalization of therapy options rely on the most accurate diagnostic procedures based on clinical pre-diagnosis and succeeding genetic confirmation. This targeted gene panel is expected to improve diagnostic and prognostic accuracy which can be applicable for personalized medicine in ADPKD. In addition, it can be applied for early diagnosis and preventive manners such as preimplantation genetic diagnosis.

## Supplementary Materials


Supplementary file 1 contains Table S1.

Supplementary file 2 contains Table S2.

Supplementary file 3 contains Table S3.
