# Supplementary material for: Targeted Next Generation Sequencing Revealed Novel Variants in the PKD1 and PKD2 Genes of Iranian Patients with Autosomal Dominant Polycystic Kidney Disease
Source: Arch Iran Med. 2022 Sep 1;25(9):600–8. doi: 10.34172/aim.2022.95 (PMC10685772; doi:10.34172/aim.2022.95)
Supplement: Supplementary file 1 — contains Table S1. [file aim-25-600-s001.pdf]

Arch Iran Med. September 2022;25(9):600-608
10.34172/aim.2022.95

Targeted Next Generation Sequencing Revealed Novel Variants in the *PKD1* and *PKD2* Genes of Iranian Patients with Autosomal Dominant Polycystic Kidney Disease

Maryam Hosseinpour, MSc[1#]; Fariba Ardalani, MSc[1#]; Marzieh Mohseni, PhD[1]; Maryam Beheshtian, MD, MPH, PhD[1]; Sanaz Arzhangi, MSc[1]; Shahrzad Ossareh, MD[2]; Hossein Najmabadi, PhD[1]; Ali Nobakht, MD[3]; Kimia Kahrizi, MD[1*]; Behrooz Broumand, MD[4*]

[1]Genetics Research Center, University of Social Welfare and Rehabilitation Sciences, Tehran, Iran
[2]Division of Nephrology, Department of Medicine, Hasheminejad Kidney Center, Iran University of Medical Sciences, Tehran, Iran
[3]Department of Nephrology, School of Medicine, Shahid Beheshti University of Medical Sciences, Tehran, Iran
[4]Pars Advanced and Minimally Invasive Medical Manners Research Center, Pars Hospital, Tehran, Iran

[#]Contributed equally as first authors

Supplementary Table 1. Full List of Genes Included in NGS Panel in this Study.

BICC1
EYA1
HNF1B
NOTCH2
PAX2
PKD1
PKD2
PKHD1
SIX5
UMOD
MUC1
PKHD1
